# COVID-19 to Green Entrepreneurial Intention: Role of Green Entrepreneurial Self-Efficacy, Optimism, Ecological Values, Social Responsibility, and Green Entrepreneurial Motivation

**DOI:** 10.3389/fpsyg.2021.732904

**Published:** 2021-10-15

**Authors:** Wenke Wang, Qilin Cao, Chaoyang Zhuo, Yunhan Mou, Zihao Pu, Yunhuan Zhou

**Affiliations:** ^1^Business School, Sichuan Normal University, Chengdu, China; ^2^Business School, Sichuan University, Chengdu, China; ^3^The People’s Bank of China School of Finance, Tsinghua University, Beijing, China; ^4^School of Public Health, Yale University, New Haven, CT, United States; ^5^Department of Statistics and Actuarial Science, The University of Hong Kong, Pokfulam, Hong Kong, SAR China

**Keywords:** green entrepreneurial intention, COVID-19, self-efficacy, optimism, ecological values, social responsibility, entrepreneurial motivation

## Abstract

This research was aimed to investigate the impact of the COVID-19 pandemic on the green entrepreneurial intention of college students through green entrepreneurial self-efficacy, optimism, ecological values, and social responsibility, as well as the mediating role of green entrepreneurial motivation. This study used structural equation model to test the hypothesis on samples of 410 Chinese colleges’ students. COVID-19 has a strong beneficial effect on green entrepreneurial self-efficacy, optimism, ecological values, and social responsibility, according to the research findings. Optimism and social responsibility also were found to have a significant positive impact on green entrepreneurial self-efficacy. Moreover, green entrepreneurial motivations moderated the relationship between optimism, ecological values, social responsibility, and green entrepreneurial intention in a positive and significant way. Finally, the findings indicate that a significant positive correlation exists between green entrepreneurial self-efficacy and optimism, as well as a significant positive correlation between ecological values and social responsibility.

## Introduction

The rapid spread of COVID-19 in the world has exerted a huge impact on the global economy, trade, and investment. As one of the most financially and socially devastating events ever to happen in history, it has affected almost every aspect of people’s lives, caused severe economic and social dislocation, and incurred tremendous financial and human resource costs, forcing the society to reexamine the way people live and work in (Barbulescu et al., 2021). Therefore, achieving economic recovery is the top priority of every country. As an important factor in promoting national economic development, entrepreneurship plays a vital role in driving economic recovery and helps to handle the COVID-19 crisis (Liguori and Winkler, 2020). Furthermore, efforts to achieve the goals of sustainable development through entrepreneurship can effectively alleviate the damage caused by COVID-19 (Anton and Rob, 2020). Apart from that, the emergence of global diseases, such as COVID-19, is inseparable from the ecological environment (Whitmee et al., 2015). As a result, it is crucial to further strengthen environmental and ecological protection on the basis of understanding relevant factors (Aldo et al., 2020). Green entrepreneurship is based on the principles of sustainability and environmental protection. It seeks to minimize the impact of entrepreneurial activities on the environment (Schaltegger, 2002), playing an irreplaceable role in maintaining social stability and achieving sustainable development of the economy, society, and environment. College students represent a vital reserve force for the country’s development, with tremendous potential for green entrepreneurship. Hence, studying the intentions of college students to start green business in times of the post-epidemic environment is extremely important to the economic recovery and development of the nation.

Ruiz-Rosa et al. (2020) found that the epidemic has caused a severe social and economic crisis and colossal uncertainty, leading to a decline in students’ intentions to start businesses. However, the post-pandemic climate for business has undergone tremendous changes, with which people’s mentality and lifestyle have also changed, resulting in a more embracing new lifestyle along with the development of network economy (Wang et al., 2020; Tang and Liang, 2021). Given that many universities must develop specific plans to promote entrepreneurship, it is essential to understand the factors that influence students’ aspirations for green entrepreneurship and to analyze the factors that influence their propensity to start green businesses by evaluating each factor involved in promoting green entrepreneurship. Through a review of literature, existing studies only discuss the relationship between COVID-19 and entrepreneurial intention, ignoring green entrepreneurship that carries out the concept of sustainable development. There is no prior research on how green entrepreneurial intentions change during a pandemic or how the epidemic affects green entrepreneurial intentions. One possible reason may be that most countries are still suffering from the pandemic. That is to say, there is no sound evidence on how exactly the COVID-19 impacts green entrepreneurship intentions. However, China is currently ushering in a post-epidemic era, with a range of policies issued by the Chinese government to support innovation and entrepreneurship. As a result, it is essential and innovative to investigate the impact of COVID-19 on college students’ intentions of green entrepreneurship.

The influence of environmental and psychological factors has always been an important topic in entrepreneurship research (Wang et al., 2019). Besides, COVID-19 has a vital impact on green entrepreneurial self-efficacy, optimism, and personality traits of green entrepreneurs. Then, we evaluated the impact of green entrepreneurial self-efficacy on green entrepreneurial intention, which is one of the main factors to effect students to carry out green entrepreneurship and establish green enterprises. The optimism variable was used to measure whether students maintained an optimistic and positive attitude toward green entrepreneurship and sustainable development in the context of COVID-19 and to investigate its impact on green entrepreneurship intention. Another factor influencing green entrepreneurial intention is personality traits of green entrepreneurs, which is relatively stable characteristic that green entrepreneurs should possess in comparison with the general population. In this study, this characteristic mainly refers to ecological values and social responsibility. Robichaud et al. (2001) believe that the entrepreneurial motivation of entrepreneurs will affect their behavioral choices and entrepreneurial outcomes. Therefore, this study also selected green entrepreneurial motivation as a mediating variable. All in all, this study focuses on the relationship between COVID-19, self-efficacy of green entrepreneurship, optimism, personal traits of green entrepreneurs, and intentions of green entrepreneurship, as well as whether the motives for green entrepreneurship can play a mediating role.

The following sections comprise this paper: an introduction, background on previous research, related theories and concepts, documents, methods, measurements, and structural models. Finally, after discussing the study results, conclusions and suggestions with theoretical, practical, and social significance are put forward, the limitations of this research and potential further research directions are summarized symptoms at presentation, physical exams, and lab results.

## Literature Review and Hypothesis Development

### COVID-19 and Green Entrepreneurial Self-Efficacy

Self-efficacy refers to an individual’s appreciation of his or her ability to successfully achieve the expected behavior. Giles et al. (2004) found in their research that self-efficacy was an important prerequisite variable of pro-social behavior. Subsequently, Hao et al. (2005) pointed out that entrepreneurial behavior also draws similar conclusions. As suggested by Bandura (2006), measurement of self-efficacy should theoretically be combined with the problem studied and the field involved. Therefore, incorporating the concept of green entrepreneurship into the concept of self-efficacy, and acknowledging that green entrepreneurial self-efficacy refers to an individual’s belief of making contributions to solve green environmental problems, reflects the individual’s confidence in his or her own efforts to improve the environment (Chu et al., 2021).

In his social cognition theory put forward in 1986, Bandura (1986) claims that individuals are affected by the surrounding environment through personal perceptions. Bandura believes that in the same environment, different people have different perceptions. Similarly, as a global pandemic, COVID-19 has various effects on individual perceptions. Therefore, perceptions of COVID-19 may influence individual behaviors. Based on historical research, risk perception has a detrimental effect on entrepreneurial self-efficacy. Gaibulloev and Sandler (2009) argued that terrorism and other violent incidents might prevent development of new businesses by reducing profits and revenues through increasing business costs. Bullough et al. (2014) studied the effect of perception: Under the unfavorable conditions of war, a decline in self-efficacy tends to resist business intentions. The findings indicate that perceived danger can reduce self-efficacy and that risk perception is negatively correlated with an individual’s entrepreneurial intentions but has a smaller effect on highly resistant individuals. In conclusion, different COVID-19 perceptions may influence individuals’ level of appreciation and confidence in their ability to achieve green entrepreneurship. Following China’s success in epidemic prevention and control, the government encourages innovation and entrepreneurship in order to promote economic recovery, at the same time, college students’ entrepreneurial awareness has been strengthened, as well as their confidence to participate in green entrepreneurship. That after, we propose the following hypothesis:

***H1a***: COVID-19 has a positive effect on green entrepreneurial self-efficacy.

### COVID-19 and Optimism

Optimism is a state of mind in which positive events are attributed to personal, long-term, and universal causes, while negative events are attributed to external, short-term, and accidental causes., while attributing negative events to external, short-term, and accidental reasons. It may help entrepreneurs in psychological overcoming objective constraints (Giacomin et al., 2013). Optimistic entrepreneurs have more positive mental states and show more positive intentions or behaviors (Bernoster et al., 2018). Some scholars have pointed out that COVID-19 not only affected people psychologically, but also brought additional anxiety to people, or reduced people’s positive and optimistic attitude (Cao et al., 2020; Tsao et al., 2020; Zurlo et al., 2020). Scholars, such as Yldrm et al. (2021), have found that COVID-19 puts people under stress and reduces optimism. Through a survey of Chinese doctors working in the COVID-19 epidemic, Zhang et al. (2020) found that optimism has a positive impact on work participation. Osimo et al. (2021) studied the effects of different psychological characteristics on mental health in the case of the COVID-19 pandemic. In terms of the psychological traits of optimism, Arslan et al. (2020) showed that coronavirus stress had a significant predictive effect on optimism, and optimism played a mediating role in the effects of coronavirus stress on adult psychological problems. Now given that the epidemic has been effectively controlled in mainland China, and the economy has been recovering steadily, cognition and awareness of COVID-19 may increase one’s level of optimism and expectations for a brighter future. Therefore, this study proposes the following hypothesis:

***H1b***: COVID-19 has a positive effect on optimism.

### COVID-19 and Personality Traits of Green Entrepreneurs

Since the 1980s, the Five-Factor Model (FFM) has served as the primary framework for studying personality traits (Goldberg, 1993). The dimensions of the FFM include extraversion, agreeableness, conscientiousness, neuroticism, and openness (Vogt and Laher, 2009). As the FFM method becomes more widely used, the amount of research on entrepreneurs’ personality traits is growing. As with other types of entrepreneurs, relative stability is a distinguishing characteristic of green entrepreneurs. Therefore, this study believes that the personality traits of green entrepreneurs are relatively stable and lasting psychological characteristics formed through the joint action of individual’s own factors and external environment, which dominate individuals’ behaviors and provide them with the ability and quality of green entrepreneurship (Tang and Zhang, 2018).

As an emerging entrepreneurial mode, green entrepreneurship is different from ordinary entrepreneurship in two aspects. First, it relies heavily on green market and green consumers (Lotfi et al., 2018). Second, it is highly dependent on policies. Since green start-ups have to take greater environmental and social responsibilities and tend to have a longer payback period, green entrepreneurial behaviors usually require the encouragement and support from policies (Nair, 2009). In addition to traditional economic issues, green entrepreneurship should also consider social responsibility and environmental issues, which establishes higher requirements for entrepreneurs in terms of ecological values and sense of social responsibility (Kirkwood and Walton, 2010; Ge et al., 2016). Therefore, the research on the personality traits of green entrepreneurs in this paper focuses on two dimensions: ecological values and social responsibilities. The emergence of global diseases, such as COVID-19, must be inseparable from the ecological environment (Whitmee et al., 2015). The rapid spread of COVID-19 and the continuous epidemic prevention efforts have made college students better understand the importance of ecological and environmental protection, and understand the social responsibilities that individuals, especially entrepreneurs, should assume when major public health emergencies occur. Therefore, the perception of COVID-19 may affect the personality traits, i.e., the ecological values and sense of social responsibility of green entrepreneurs. This study proposes the following hypotheses:

***H1c***: COVID-19 has a positive effect on ecological values.

***H1d***: COVID-19 has a positive effect on social responsibility.

### Green Entrepreneurial Self-Efficacy and Optimism

Do green entrepreneurial self-efficacy and optimism affect each other? According to previous studies, people with high self-efficacy tend to show better stress tolerance and have a more positive psychological state (Neneh, 2020). On the other hand, Martínez-González et al. (2019) found that subjective factors (beliefs, social norms, and values) trigger a series of events that influence action variables (motivation, self-efficacy, and intent). Optimism is also a subjective variable, and events triggered by optimism will affect self-efficacy. Hmieleski and Baron (2008) found that optimism and environmental vitality are potential mediating factors of the impact of entrepreneurial self-efficacy on corporate performance. Based on the experimental results, it is verified that these factors do mediate the impact of entrepreneurial self-efficacy. Tong and Miao (2019) found that in the learning process of college students, optimism plays a partial mediating role between self-efficacy and academic performance. Chu et al. (2020) found that emotional competence has a significant positive impact on entrepreneurial self-efficacy in the process of studying entrepreneurial intentions of Chinese college students, and entrepreneurial self-efficacy plays a mediating role between emotional competence and entrepreneurial intentions. In conclusion, people with higher self-efficacy in green entrepreneurship maintained a more optimistic attitude toward entrepreneurship. Positive and optimistic people can be more confident in completing their business, and they have higher expectations of success. Based on the above literature, this paper proposes the following hypothesis:

***H2***: Green entrepreneurial self-efficacy and optimism have a positive effect on each other.

### Ecological Values and Sense of Social Responsibility

Values refer to a series of standards or goals that play a guiding role in people’s life, attitudes, and behaviors. Individuals’ perspectives on their relationship with the environment, as well as the society, determine their value choices (Demchenko, 2008). Ecological values are critical standards and guidelines that people use to manage such relationships. At present, the research on ecological values mainly focuses on two issues. First, let us consider the structural dimension of ecological values. Dunlap (1980) believed ecological values were monodimensional, whereas Amburgey and Thoman (2012) presumed ecological values are multidimensional. Second, the factors affecting ecological values. Foreign scholars have found through a large number of empirical studies that demographic factors, such as education background, age, and gender, have a great impact on an individual’s ecological values (Steg et al., 2011).

In addition to ecological values, another dimension of green entrepreneurial personality is social responsibility. Cohen and Winn (2007) pointed out that green entrepreneurship was a multiple-target behavior carried out in an uncertain environment. Its core is to develop a green market with innovation, while also taking on corresponding social and ecological responsibilities, implying that individuals are willing to take on corresponding social responsibilities at startup while considering the dual roles of ecological construction and entrepreneurship. Many social demands cannot be covered or met by the government due to the nature of green products as public goods, according to some case studies. Hence, the sense of social responsibility of an entrepreneurial team is crucial for green entrepreneurship (Sharir and Lerner, 2006). When constructing the conceptual model of green entrepreneurship, Abrahamsson (2007) also considers the social mission of the person in charge as a key factor affecting the development of an enterprise. As a result, a sense of social responsibility (social mission, primarily referring to a sense of responsibility related to green areas) is critical in the formation of green entrepreneurial intentions, if not the entire process of green entrepreneurship. Entrepreneurs with a sense of social responsibility are more likely to stick to their green business goals and not give up easily when faced with challenges. It can be concluded based on FFM that ecological values and social responsibilities, as two dimensions of the personality traits of green entrepreneurs, are the same type of variables that may have effects on each other. Therefore, this paper proposes the following hypothesis:

***H3***: Ecological values and social responsibilities have a positive effect on each other.

### Green Entrepreneurial Self-Efficacy, Green Entrepreneurial Motivation, and Green Entrepreneurship Intentions

**Figure 1:**
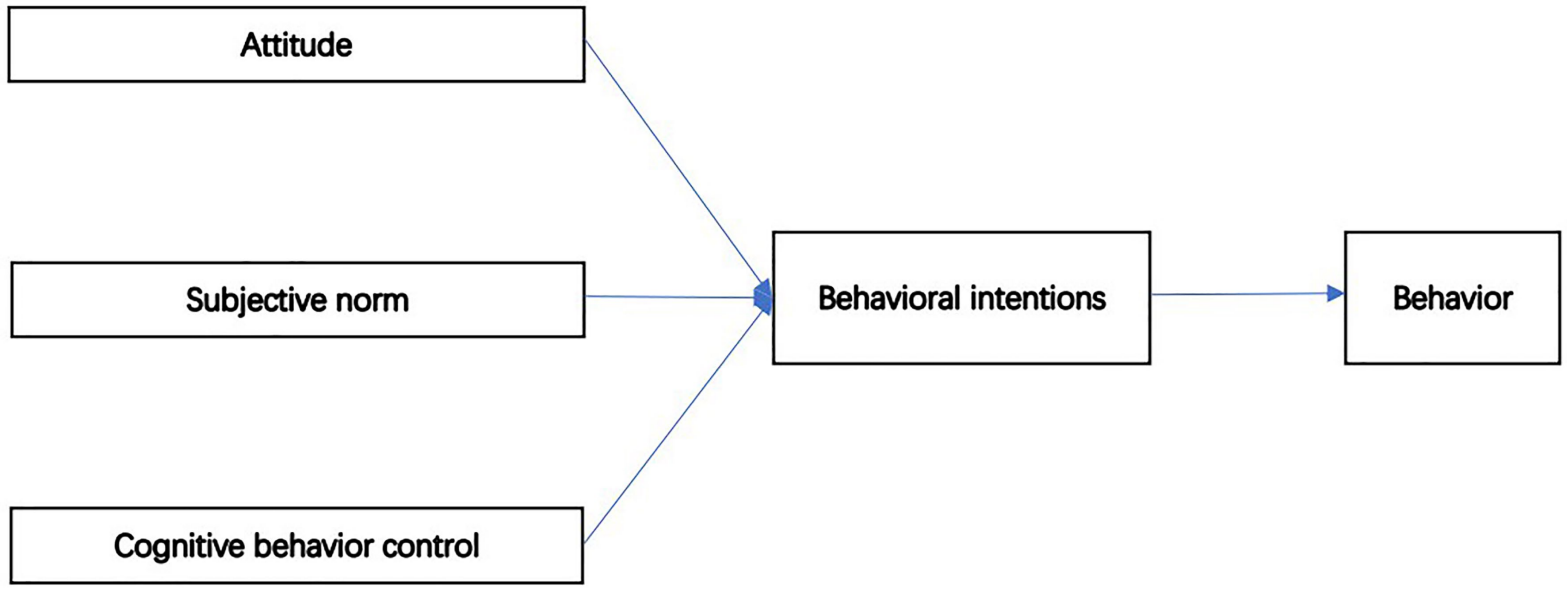
TPB model.

Bird (1988) defines intention as “a state of mind directing a person’s attention (and therefore experience and action) toward a specific object (goal) or a path to achieve something (means).” Results based on psychological studies show that measuring one’s intention for a behavior can predict his or her future behavior to a certain extent (Armitage and Conner, 2001). Specifically, Ajzen (1991) associated entrepreneurial intentions with entrepreneurial behaviors in 1991 for the first time. After that, Shapero and Sokol (1982) developed different models to study intentions in the field of entrepreneurship. Among them, the research results of the Theory of Planned Behavior (TPB) have provided great help and support for the study of entrepreneurial behavior, TPB model has become an effective approach in the study of entrepreneurial behaviors and intentions (Lortie and Castogiovanni, 2015). Figure 1 shows the TPB model.

Based on the theories, such as TPB, Mahoney and Micheal, (2005) believe that entrepreneurial intention is the most important factor affecting entrepreneurial behavior. According to previous studies, the higher a person’s level of entrepreneurial self-efficacy, the stronger his or her entrepreneurial intention (Chu et al., 2020). Ding and Ding (2011) found that entrepreneurial self-efficacy has a strong predictive effect on the occurrence of entrepreneurial intention and entrepreneurial behavior. Tu and Wang (2017) found that there was a significant positive correlation between college students’ general self-efficacy and entrepreneurial intention. Compared with general entrepreneurship, green entrepreneurship faces greater challenges in the investment and innovation of green technology. When entrepreneurs are full of confidence in their green entrepreneurship ability and have high green entrepreneurship self-efficacy, they are more likely to have strong green entrepreneurship intentions (Alvarez-Risco et al., 2021).

Entrepreneurial motivation refers to the internal driving force to achieve a specific vision and goal through entrepreneurship according to individual needs. The difference in motivation directly affects the entrepreneurial process and performance (Carsrud and Braennback, 2011). Green entrepreneurship motivation is the key incentive to promote green entrepreneurship. Entrepreneurial motivation, according to Robichaud et al. (2001), is the goal that entrepreneurs pursue using their business rights, which directly influences their behavior choices, while behaviors determine the success of entrepreneurial activities. Therefore, their entrepreneurial motivation affected the entrepreneurial results indirectly. It can be said that entrepreneurial motivation is a willingness of individuals to participate in entrepreneurial activities (Hassan et al., 2021). The differences in intentions will influence a range of individual behaviors in the dynamic process of pursuing entrepreneurial opportunities. Reynolds et al. (2005) believe that entrepreneurial motivation can be roughly divided into two types: opportunistic and need-based motivation. Opportunistic entrepreneurs pursue entrepreneurial value, while need-based entrepreneurs pursue material benefits. Similar to opportunistic entrepreneurs, green entrepreneurs seek a market demand for a green product or service, that is, to create commodity value in the ecological field. Shi et al. (2016) divided entrepreneurial motivation into fame and profit motivation and opportunity motivation, and the results showed that both types of motivation positively influenced entrepreneurial intention. In conclusion, green entrepreneurial self-efficacy will affect the judgment of entrepreneurial feasibility, and green entrepreneurial intention will be significantly improved if it is combined with the internal or external impetus of green entrepreneurial motivation. Consequently, the following hypotheses are proposed as:

***H4***: Green entrepreneurial self-efficacy has a positive effect on green entrepreneurial intentions.

***H8a***: Green entrepreneurial self-efficacy has an impact on green entrepreneurial intentions through green entrepreneurial motivation.

### Optimism, Green Entrepreneurial Motivation, and Green Entrepreneurial Intentions

Optimistic attitude tends to enhance an entrepreneur’s self-confidence and entrepreneurial intentions. Dushnitsky (2010) believe that optimism is the key factor of entrepreneurship. Optimistic students are usually full of confidence (Lee et al., 2018), more expect to achieve positive results in entrepreneurship, and show higher business intention (Giacomin et al., 2013). Trevelyan (2008) found a strong correlation between optimism, overconfidence, and entrepreneurship based on their research, while Bernoster et al. (2018) claim that optimism positively affects entrepreneurial intentions. When students are optimistic about entrepreneurship, they have higher expectations for the process and results of entrepreneurship and are more willing to choose green entrepreneurship.

On the other hand, optimistic entrepreneurs are more motivated to start their own businesses. Taormina and Lao (2007) studied Chinese entrepreneurs based on the Push and Pull Theory and believed that entrepreneurial motivation could be measured by four variables: optimism, need for achievement, social network, and business environment. Optimism can enhance the inner driving force of green entrepreneurial motivation and influence the willingness of individuals to participate in green entrepreneurial activities. Therefore, optimism may positively affect the entrepreneurial intentions of green entrepreneurs by enhancing their entrepreneurial motivation and influencing their entrepreneurial behavior choices. This study proposes the following hypotheses:

***H5***: Optimism has a positive effect on green entrepreneurial intentions.

***H8b***: Optimism has an effect on green entrepreneurial intentions through green entrepreneurial motivation.

### Personality Traits of Green Entrepreneurs, Green Entrepreneurial Motivation, and Green Entrepreneurial Intentions

On the basis of an in-depth study of the previous literature, Chi (2010) proposed the theory of quality of entrepreneurs. He contended that the quality of entrepreneurs was the core of entrepreneurship and directly affected the formation of entrepreneurial intentions. Tang and Zhang (2018) investigated the direct impact of creative personality traits on entrepreneurial self-efficacy and entrepreneurial intention, and the mediating role of entrepreneurial self-efficacy between creative personality traits and entrepreneurial intention, and found that creative personality traits can improve entrepreneurial intention. Green entrepreneurial personality is the sum of individuals’ long-term and relatively important psychological characteristics. It can control individuals’ entrepreneurial behavior, affect their entrepreneurial spirit, and directly affect individuals’ intention to participate in green entrepreneurship (Qazi et al., 2020).

In terms of entrepreneurial motivation, Dou and Luo (2011) summarized entrepreneurial motivation according to the hierarchical theory of needs. They suggest that entrepreneurial motivation can be divided into two aspects: the stimulus of economic needs and the stimulus of social needs. The former is based on physiological needs and safety needs. These two hierarchical needs are the essential needs of human beings and must be satisfied by economic means. And the original motivation that drives individuals to start businesses is to obtain economic benefits. The latter is based on self-esteem and self-actualization needs, including a sense of accomplishment (Zhao et al., 2010), respect, personal fulfillment, confidence, and other needs that should be acknowledged by society or others. In addition, the motivation of green entrepreneurs is also closely related to their personal value orientation. The original intention of many green entrepreneurs is to spread their green values, improve consumers’ awareness of green consumption, and achieve the goal of green entrepreneurship (Kirkwood and Walton, 2010). Therefore, based on social needs, realization of ecological values and the sense of social responsibilities will affect green entrepreneurial motivation, which in turn influences the behaviors and choices of entrepreneurs, and even the entrepreneurial intentions. In this study, the following hypotheses are proposed as:

***H6***: Ecological values have a positive effect on green entrepreneurial intentions.

***H7***: Sense of social responsibility has a positive effect on green entrepreneurial intentions.

***H8c***: Ecological values have an effect on green entrepreneurial intentions through green entrepreneurial motivation.

***H8d***: Sense of social responsibility has an effect on green entrepreneurial intentions through green entrepreneurial motivation.

### Conceptual Model

**Figure 2:**
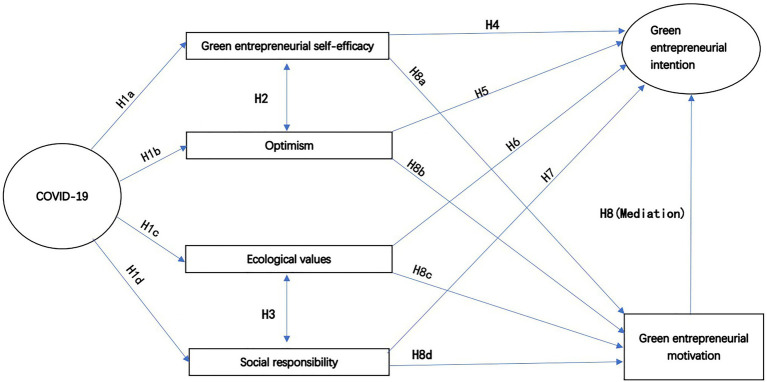
Research framework.

In this study, we attempt to clarify the relationship between COVID-19 and green entrepreneurial self-efficacy, optimism, and green entrepreneurial personality traits (ecological values and social responsibility), as well as their impact on green entrepreneurial intentions. Besides that, we want to understand how the psychological factors mentioned above influence the willingness of green entrepreneurs to engage in green entrepreneurship *via* green entrepreneurial motivation. In order to reach those goals, we conceptualized a model that includes the variables above. Figure 2 shows our research framework.

## Materials and Methods

### Pilot Testing

A preliminary questionnaire was developed based on the validated scale. Forty students from business schools of different universities in Chengdu, Sichuan Province, China were randomly selected to conduct a pilot testing. In this study, a combination of various scales was formed to output a temporary scale (in Chinese) for green entrepreneurial intentions under the COVID-19 pandemic. After analyzing the content, reliability, and validity of the initial questionnaire, we modified part of the initial questionnaire and determined the final form of the questionnaire, which consisted of 32 items. The respondents were asked to give their opinions in scale options following the 5-point Likert format, with 1 to 5 indicating “entirely disagree,” “disagree,” “unsure,” “agree,” and “entirely agree” in turn. Table A1 shows the content of the questionnaire.

### Population and Sampling Technique

The formal questionnaire uses a random sampling method to conduct a questionnaire survey of college students from different universities in China. After respondents were informed of the anonymity of their identities, which obtained full consent, the questionnaires were distributed and 410 valid questionnaires were recovered. Among them, men completed 197 copies and women completed 213 copies. A total of 206 of the respondents were under the age of 20, 193 respondents were 21–25years old, and 11 respondents were 26years old or older. There were 352 undergraduates, 53 postgraduates, and 5 doctoral students among the respondents.

### Measures

#### Green Entrepreneurial Self-Efficacy

The questionnaire items in the section of green entrepreneurial self-efficacy are based on three particular items designed by Hockerts’s (2015) in their research, combining the characteristics of green entrepreneurship with Tang’s, 2019 research on green entrepreneurial self-efficacy. A sample item is as: “I can find a way to help solve environmental problems.” A five-point scale was used, with 1 indicating complete disagreement and 5 indicating complete agreement.

#### Optimism

The optimism section contains three items derived from Zhang’s et al. (2010) scale, which was used in Liu’s (2017) research to examine the relationship between optimism and entrepreneurship and produced positive results. A sample item is as: “Most of the time, I am high-spirited.” A five-point scale was used, with 1 indicating complete disagreement and 5 indicating complete agreement.

#### Personality Traits of Green Entrepreneurs

The ecological values section contains four items that refer to a 2007 WVS (World Values Survey) questionnaire on the Chinese public’s ecological environment values. This questionnaire covered the concepts of environmental stewardship, business commitment to environmental protection, economic and environmental relations, and awareness of environmental problems. Seven items are included in the four dimensions of environmental satisfaction. They are used to assess college students’ ecological values. Four of them are relevant to green entrepreneurship, which was the subject of this study. A sample item is as: “I am willing to give out part of the income if I am sure that the money can be used to prevent and control environmental pollution.”

The social responsibility sense scale section contains three items that were comprehensively selected using the scales of Hockerts’s (2015), Wang and Li (2017), Du and Xie (2012). A sample item is as: “After a major disaster, I tend to donate money or useful goods.”

#### Green Entrepreneurial Motivation

The green entrepreneurial motivation section contains six items derived from the scales of Zhang and Lei (2012); Zhang (2019). Among them, Zhang (2019) studied the mediating effect of green entrepreneurial motivation on green entrepreneurial intention based on this scale, and this effect was proved to be significant. A five-point scale was used, with 1 indicating complete disagreement and 5 indicating complete agreement. A sample item in green entrepreneurial motivation section is “I would like to start a green enterprise in order to solve employment problems.”

#### Green Entrepreneurial Intentions

The green entrepreneurship intentions section consists of five items. As Zhang (2019) referred to Linan and Chen (2009)’s entrepreneurial intentions scale and improved it by adding the features of green entrepreneurial intentions. Based on this improved version, this research further integrated the green entrepreneurial intentions scales suggested by Duan and Du (2012); Wang and Li (2017). A sample item in green entrepreneurial intentions section is as: “I have seriously considered things about green entrepreneurship.” All items were evaluated on a five-point scale, with 1 indicating complete disagreement and 5 indicating complete agreement.

#### COVID-19 Perception

The COVID-19 perception section contains six items. Three items have been improved based on the research of Tian (2005); Diclemente et al. (2011); Zhu et al. (2020); and the “SARS Social Psychological Survey Questionnaire” developed and compiled by the Chinese Academy of Sciences’ Institute of Psychology. The rest three items were improved on GAD-7 (International Most Common Anxiety Disorder Table) by combining them with COVID-19. In fact, Zurlo et al. (2020) have proved that GAD-7 could be effectively applied to COVID-19. A sample item is as: “During COVID-19, I tend to worry too much.” A five-point scale was used, with 1 indicating complete disagreement and 5 indicating complete agreement.

## Results

### Data Analysis and Results

In this study, the SPSS 22.0 (IMB Company, Armonk, New York, United States) and the AMOS 22.0 (IBM Corp., Armonk, NY, United States) were used for data analysis. SPSS can not only perform basic descriptive statistical analysis on the results of the questionnaire survey, but also perform reliability and validity tests. AMOS can be used to construct and calculate structural equations through visual operations, and give appropriate information for the constructed structural equations, like suggestions on the adjustment of equations.

### Measurement Model Analysis

Before modeling with structural equation, we first evaluate the reliability and validity of our questionnaire survey. Cronbach’s alpha is widely used to measure the degree of reliability. In this study, Cronbach’s coefficient was obtained through reliability analysis in SPSS, and reliability tests were conducted for both individual factors and the overall model. The results are shown in Table 1. The six Cronbach alpha coefficients are all greater than 0.9, except one, which is still greater than 0.85, indicating that each variable has good internal consistency. The Cronbach alpha coefficient obtained by the overall reliability test of the scale designed is 0.961, showing the overall reliability of the scale is also very good.

**Table 1:** The results of reliability test.

Variable	Cronbach α	No. of Items
Green entrepreneurial self-efficacy	0.908	3
Optimism	0.888	3
Green entrepreneurial personality traits: Ecological values	0.930	4
Green entrepreneurial personality traits: Social responsibility sense	0.901	3
Green entrepreneurial motivation	0.938	6
COVID-19 perception	0.953	5
Green entrepreneurial intentions	0.924	5

Validity refers to how accurate the scale can be used to measure the variable of interests. In short, it means the degree of discrimination between latent variables. Validity can be considered from two aspects: content validity and structure validity. The measurement scales used in this study are all mature ones from around the world, and our questionnaires are developed using professional translation and expert opinion, ensuring high content validity. In terms of structural validity, the results of the validity test are shown in Tables 2–4. In Table 2, the KMO value is 0.930, higher than 0.8 and value of p of Bartlett Ball test is lower than 0.001, indicating that it is suitable to conduct factor analysis on our dataset to assess the validity. Besides that, we did confirmatory factor analysis (CFA) and obtained 7 factors consisting of weighted items based on questionnaire data. And we expected that each factor obtained by CFA is according with one specific defined psychological factor, for instance, factor 5 is corresponding to green entrepreneurial self-efficacy (Z1). Other correspondences between factors and defined psychological factors are presented in the last row of Table 3. Additionally, we created a column in Table 3 to denote the meanings of each row; for instance, Z11 is one of the questions about green entrepreneurial self-efficacy. As shown in Table 3, the common degree values of all research items are greater than 0.4, indicating that the information contained in the research items can be extracted effectively. Likewise, the cumulative variance explanation rate after rotation is 74.201% which is greater than 50%, and the amount of information contained in research items can be extracted effectively as well. Moreover, the relationship between factor loading coefficient confirmation factor (dimension) and research variables is also satisfactory. As can be seen from the CFA analysis results in Table 4, each factor has a CR value greater than 0.8, indicating that the latent variables have a high degree of internal consistency. Also, all AVE values are greater than 0.5, indicating that latent variables are capable of explaining a substantial amount of variation. Thus, our study has a high level of validity in terms of both content and structure.

**Table 2:** KMO and Bartlett Ball test.

KMO Value		0.93
Bartlett Ball test	Approximate chi-square	8040.806
	df	406
	value of p	< 0.001

**Table 3:** Factor loading coefficient and variance interpretation rate.

Item	Factor loading coefficient							Common degree (common factor variance)	Meanings of the item
	Factor1	Factor2	Factor3	Factor4	Factor5	Factor6	Factor7		
Z11	0.114	0.107	0.081	0.201	**0.811**	0.133	0.202	0.788	Questions related to green entrepreneurial self-efficacy
Z12	0.265	0.168	0.063	0.208	**0.717**	0.174	0.088	0.697	
Z13	0.107	0.079	0.028	0.232	**0.818**	0.144	0.110	0.775	
Z21	0.173	0.185	0.097	0.141	0.104	**0.788**	0.093	0.733	Questions related to optimism
Z22	0.225	0.115	0.017	0.202	0.185	**0.732**	0.056	0.677	
Z23	0.156	0.181	0.043	0.143	0.145	**0.794**	0.143	0.752	
Z31	0.195	0.163	0.116	**0.702**	0.101	0.200	0.174	0.652	Questions related to ecological values
Z32	0.216	0.192	0.133	**0.714**	0.164	0.174	0.059	0.671	
Z33	0.119	0.026	0.085	**0.784**	0.191	0.072	0.148	0.700	
Z34	0.148	0.026	0.028	**0.738**	0.225	0.116	0.220	0.681	
Z41	0.154	0.106	0.113	0.133	0.211	0.099	**0.785**	0.737	Questions related to social responsibility sense
Z42	0.240	0.275	0.090	0.194	0.115	0.160	**0.711**	0.723	
Z43	0.212	0.149	0.086	0.299	0.095	0.072	**0.760**	0.756	
M1	**0.681**	0.366	0.085	0.240	0.141	0.172	0.152	0.735	Questions related to green entrepreneurial motivation
M2	**0.736**	0.275	0.131	0.181	0.114	0.184	0.100	0.724	
M3	**0.766**	0.193	0.168	−0.001	0.050	0.112	0.120	0.681	
M4	**0.827**	0.245	0.102	0.077	0.108	0.124	0.181	0.819	
M5	**0.798**	0.247	0.051	0.246	0.102	0.121	0.127	0.801	
M6	**0.800**	0.176	0.040	0.229	0.168	0.122	0.112	0.780	
Y1	**0.412**	**0.706**	0.070	0.184	0.176	0.100	0.110	0.759	Questions related to green entrepreneurial intentions
Y2	0.270	**0.823**	0.138	0.089	0.099	0.115	0.114	0.813	
Y3	0.296	**0.808**	0.083	0.138	0.062	0.105	0.099	0.792	
Y4	0.222	**0.823**	0.183	0.068	0.082	0.153	0.171	0.824	
Y5	0.190	**0.860**	0.139	0.016	0.061	0.157	0.102	0.833	
COVID1	0.057	0.214	**0.742**	0.082	−0.012	0.096	0.165	0.643	Questions related to COVID-19 perception
COVID2	0.011	0.032	**0.802**	0.039	0.047	0.035	0.071	0.655	
COVID3	0.117	0.065	**0.857**	0.105	0.057	−0.012	0.009	0.766	
COVID4	0.136	0.095	**0.864**	0.112	0.087	0.019	0.002	0.795	
COVID5	0.103	0.105	**0.854**	0.002	0.001	0.047	0.044	0.755	
Factor is corresponding to	M	Y	COVID	Z3	Z1	Z2	Z4		

Bold values in table 3 means the maximum value of each row. To be more specific, it means the item of each row is the most likely to belong to which Factor, which has been described in Measure Model Analysis part.

**Table 4:** CR and AVE values of each factor.

Factor	AVE	CR
Factor1	0.621	0.831
Factor2	0.580	0.805
Factor3	0.552	0.831
Factor4	0.592	0.812
Factor5	0.685	0.929
Factor6	0.744	0.935
Factor7	0.653	0.902

### Structural Model Analysis

The structural equation model was implemented in this study using the AMOS software. The indexes that evaluate the fitness of the model are shown in Table 5. From the overall evaluation indicators in Table 5, it can be seen that the structural equation constructed in this paper has reached the reference standard in the chi-square degree of freedom ratio, RMSEA, CFI, NNFI, TLI, IFI, and other indicators, showing that the data fit excellently in the structural equation of this paper.

**Table 5:** Fitness of model.

Index name	Judgment standard	Result of model
Chi-square degree of freedom ratio *χ*^2^/df	< 3	2.783
RMSEA	< 0.10	0.066
CFI	> 0.9	0.918
NNFI	> 0.9	0.908
TLI	> 0.9	0.908
IFI	> 0.9	0.918

**Figure 3:**
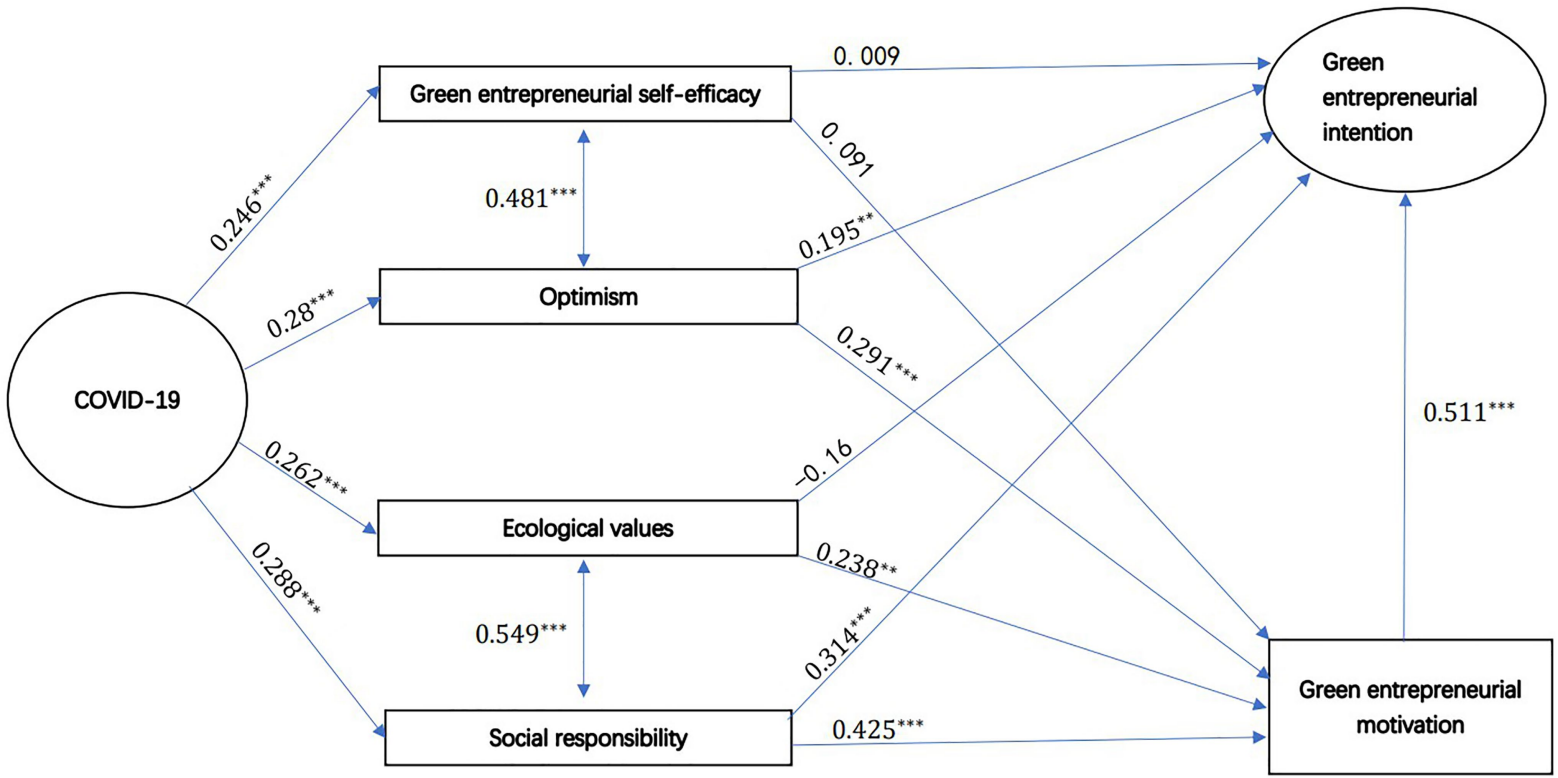
Structure model.

Table 6 summarizes the test results for each hypothesis. In each row, we gave unstandardized and standardized regression coefficient of each estimation. And standard error, CR-values, and value of p are given in Table 6 which indicates the coefficient’s significance. As well as Figure 3 illustrates the structure of this model along with the results of each path. The values associated with edges in this figure are unstandardized regression coefficients, which had been presented in Table 6. In addition, as there are some abbreviations used in this methodology part, we created a list for those abbreviations in Table 7 for reference.

**Table 6:** Structural model path estimation.

A	Relationship	B	Unstandardized regression coefficient	*Standard Error*	CR-Value	*p*	Standardized regression coefficient
Z1	→	M	0.091	0.063	1.443	0.149	0.086
Z1	→	Y	0.009	0.063	0.149	0.882	0.008
Z2	→	M	0.291	0.060	4.820	0.000	0.302
Z2	→	Y	0.195	0.062	3.114	0.002	0.191
Z3	→	M	0.238	0.089	2.655	0.008	0.183
Z3	→	Y	−0.160	0.090	−1.783	0.075	−0.117
Z4	→	M	0.425	0.089	4.803	0.000	0.344
Z4	→	Y	0.314	0.092	3.414	0.001	0.240
M	→	Y	0.511	0.065	7.835	0.000	0.484
COVID	→	Z1	0.246	0.062	3.945	0.000	0.225
COVID	→	Z2	0.280	0.070	3.997	0.000	0.233
COVID	→	Z3	0.262	0.052	5.047	0.000	0.294
COVID	→	Z4	0.288	0.055	5.198	0.000	0.307
Z1	mutual	Z2	0.236	0.033	7.104	0.000	0.481
Z3	mutual	Z4	0.171	0.023	7.578	0.000	0.549

**Table 7:** List for abbreviations.

Abbreviations	Referring To
Z1	Green entrepreneurial self-efficacy
Z2	Optimism
Z3	Green entrepreneurial personality traits: Ecological values
Z4	Green entrepreneurial personality traits: Social responsibility sense
M	Green entrepreneurial motivation
Y	Green entrepreneurial intentions
COVID	COVID-19 (Corona Virus Disease 2019) perception
df	Degree of Freedom
AVE	Average Variance Extracted
CR	Capability Ratio
RMSEA	Root Mean Square Error of Approximation
CFI	Comparative Fit Index
NNFI	Non-Normed Fit Index
TLI	Tucker Lewis Index
IFI	Incremental Fit Index
TPB	Theory of Planned Behavior

Discussing H1, this article proposes that COVID-19 is positively affecting green entrepreneurial self-efficacy, optimism, ecological values, and social responsibility. The model results indicate that the effect of COVID-19 on them is all positive, and the significance level is 0.01 or higher, indicating that the effect of COVID-19 on them is of practical significance. Among them, COVID-19 has a strong positive relationship to optimism (H1b, *β*=0.233, *p*<0.001), to the characteristics of social responsibility (H1d, *β*=0.307, *p*<0.001), to characteristics of the positive effects of ecological values (H1c, *β*=0.294, *p*<0.001), and to green entrepreneurial self-efficacy (H1a, *β*=0.225, *p*<0.001). In conclusion, hypothesis H1 holds true.

Considering H2 and H3, this study estimates the interrelationship between two independent variables, the interrelationship between green entrepreneurial self-efficacy, and optimism (H2, *β*=0.481, *p*<0.001), as well as ecological values and social responsibility (H3, *β*=0.549, *p*<0.001). The two sets of correlations are both positive and relatively strong, which is consistent with our inference from the psychological professional perspective described in the previous article.

Regarding H4, this article assumes that the self-efficacy of green entrepreneurship has a positive impact on the willingness associated with green entrepreneurship. Regarding H8a, this article assumes that green entrepreneurial self-efficacy positively affects green entrepreneurial intentions through green entrepreneurial motivation. The model’s results do not match the hypotheses. To be more specific, as for the influence of green entrepreneurial self-efficacy on green entrepreneurial intentions, whether it is indirectly affected by intermediary green entrepreneurial motivation (H8a, *β*=0.086, *p*=0.149) or directly affects green entrepreneurial intentions (H4, *β*=0.008, *p*=0.882), the significance of the paths is above 0.1, indicating that the influence relationship is not significant. Therefore, based on the results of our questionnaire survey, we believe that hypotheses H4 and H8a are not valid.

Concerning H5, this article assumes that optimism has a positive impact on green entrepreneurial intentions. Regarding H8b, this article assumes that optimism indirectly and positively affects green entrepreneurial intentions through green entrepreneurial motivation. The results show that significance level of optimism’s impact on green entrepreneurial intentions, whether it is a direct impact (H5, *β*=0.191, *p*=0.002) or an indirect impact (H8b, *β*=0.302, *p*<0.001), has reached 0.01 or greater, indicating that optimism can affect green entrepreneurial intentions either indirectly or directly through an intermediary relationship with green entrepreneurial motivation. Hypothesis H5 and H8b are both valid.

Regarding H6, this article assumes that ecological values have a positive impact on green entrepreneurial intentions. Considering H8c, this article implies that ecological values influence green entrepreneurial intentions indirectly and positively *via* green entrepreneurial motivation. The results show that ecological values’ indirect effect on green entrepreneurial intentions through the intermediary relationship (H8c, *β*=0.183, *p*=0.008) is highly significant, with value of *p* is below 0.01. However, the path significance of its direct impact (H6, *β*=−0.117, *p*=0.075) failed to reach 0.05. Therefore, this article believes that the hypothesis H6 is not valid, and H8c is valid. According to standardized coefficients, students with stronger ecological values are more willing to start a green business.

Regarding H7, this article assumes that the sense of responsibility has a positive impact on green entrepreneurial intentions. Regarding H8d, this article assumes that the sense of responsibility will indirectly and positively affect the willingness of green entrepreneurship through the motivation of green entrepreneurship. The results show that the significance of social responsibility’s impact on green entrepreneurial intentions, whether it is a direct impact (H7, *β*=0.24, *p*=0.001) or an indirect impact (H8d, *β*=0.344, *p*<0.001), has reached 0.01 or higher, indicating that social responsibility can affect green entrepreneurial intentions through intermediary green entrepreneurial motivation or direct effects, which means hypotheses H7 and H8d are both valid.

## Conclusion and Recommendations

### Conclusion

Based on the findings so far, the following conclusions can be drawn. Perception of COVID-19 has a positive impact on green entrepreneurial self-efficacy (H1a), ecological values (H1c), and social responsibility (H1d). In other words, perception of COVID-19 can encourage college students to improve green entrepreneurial self-efficacy and to promote the formation of ecological values and social responsibility. Following the COVID-19 epidemic, college students developed a stronger sense of environmental stewardship and social responsibility. As a result of their involvement in the fight against COVID-19, the students surveyed improved their perception and awareness, which is consistent with the results of this questionnaire survey.

The effect of COVID-19 on optimism is positive (H1b), which may be controversial to our intuition. But in fact, the majority of people surveyed in this study is college students in mainland China, and the survey was conducted in the first quarter of 2021. By this time, the situation in mainland China has been greatly improved after rigorous control of COVID-19 in 2020, and vaccination campaigns have been undertaken worldwide. China’s macroeconomic indicators are also satisfying. According to the data released by the National Bureau of Statistics of China, the GDP growth rate in the first quarter reached 18.3%. Additionally, because mainland China’s universities have almost completely restored conventional offline teaching in the spring term of 2021, the increasing optimism of those surveyed is due to the affirmation of epidemic control and steady economic recovery, not to the outbreak of the epidemic. Therefore, the conclusion is in fact in line with the actual situation.

Green entrepreneurial self-efficacy has no significant effect on green entrepreneurial intentions either directly (H4) or through the indirect effect of green entrepreneurial motivation (H8a). In other words, green entrepreneurial self-efficacy has no significant impact on green entrepreneurial intentions.

Optimism can directly (H5) or indirectly (H8b) influence green entrepreneurial intentions through green entrepreneurial motivation. This is consistent with the research conclusions of Bernoster et al. (2018). Whether it is ordinary entrepreneurship or the green entrepreneurship discussed in this article, certain risks exist. Optimistic attitude does have a very important effect on individual’s entrepreneurial intentions and it can aid in the development of entrepreneurial intentions. This study established this conclusion from a green entrepreneurship perspective.

Although ecological values cannot directly influence green entrepreneurial intentions(H6), they can have an indirect effect on green entrepreneurial intentions (H8c) through green entrepreneurial motivation. Green entrepreneurs with a personality trait of strong ecological value will have a higher level of green entrepreneurial motivation and intentions of green entrepreneurship. However, ecological values have no significant direct impact on green entrepreneurial intentions.

A sense of social responsibility can have a direct (H7) or indirect (H8d) effect on green entrepreneurial intentions. The intensity of effect or direct impact of a sense of social responsibility on green entrepreneurial intentions through green entrepreneurial motivation is higher than that of other established factors, because a sense of social responsibility is an important difference between green entrepreneurship and ordinary entrepreneurship. At the very beginning of starting a business, green entrepreneurship can be regarded as aiming to make contributions to society. Therefore, a greater sense of social responsibility has a very strong impact on green entrepreneurial intentions.

In addition, there is a distinct interrelationship and strong correlation between green entrepreneurial self-efficacy and optimism (H2), which is consistent with the results of the literature (Baron, 2010; Tong and Miao, 2019; Chu et al., 2020) There is also a significant interrelationship and correlation between ecological values and a sense of social responsibility (H3). Ecological values and a sense of social responsibility can have a positive mutual promotion effect.

### Recommendations

First of all, more emphasis should be put on cultivation of college students’ green entrepreneurial intentions by focusing on the aspects of optimism, ecological values, and a sense of social responsibility. The findings of this study indicate that these three psychological factors have a significant and positive effect on and facilitate green entrepreneurial intentions. For instance, from a psychological and sociological perspective, a sense of social responsibility is a necessary component of green entrepreneurship. In green entrepreneurship, entrepreneurs need to devote a considerable amount of their energy to “helping and giving back to society,” which is often caused by a sense of responsibility. Ecological values follow a similar pattern. Green entrepreneurship is closely related to ecology, and good ecological values will naturally have a significant effect on the cultivation of green entrepreneurial intentions. A specific course or specific publicity does not result in the strengthening and construction of psychological factors, such as a sense of social responsibility, ecological values, and optimism. This study demonstrates that COVID-19 epidemic has a certain positive effect on the sense of social responsibility, ecological values, and optimism of the college students surveyed and can also enhance their sense of social responsibility to some extent. In fact, this is probably due to the influence of various factors in epidemic prevention and control, such as the strong sense of social responsibility of healthcare workers, public health workers, and volunteers, as well as the ecological and environmental issues reflected during the COVID-19 epidemic and related publicity. Our research examines the effect of psychological factors, such as optimism, ecological values, and social responsibility on college students’ intentions to pursue green entrepreneurship. As a result, there are numerous opportunities to cultivate college students’ intentions to start green businesses, and capitalizing on these opportunities is very essential.

Second, attention should be paid to the interrelationship between different psychological factors. We examined the relationship between two sets of psychological factors in this study: self-efficacy and optimism, as well as ecological values and a sense of social responsibility. There is a significant correlation between the two factors of each group, and the correlation coefficient of these two groups is around 0.5, indicating a strong positive correlation. This suggests that, when paying attention to the psychological factors related to green entrepreneurial intentions, the correlation between psychological factors should also be taken into consideration. In corresponding training or publicity activities, such correlation can also be fully considered to make cultivation and construction of green entrepreneurial intentions more efficient. For example, on cultivation of ecological values and a sense of social responsibility, due to their stronger positive correlation, propaganda and education of these factors can be carried out at the same time, which can not only save the cost of publicity, but also make the propaganda more effective, as well as facilitate formation of green entrepreneurial intentions. In terms of self-efficacy and optimism, although self-efficacy itself has no significant impact on green entrepreneurial intentions, it is strongly correlated with optimism. Therefore, self-efficacy should be properly considered in propaganda or cultivation rather than be totally abandoned.

Furthermore, the difference between green entrepreneurial motivation and green entrepreneurial intentions should be noted and taken into account in formulation and implementation of practical policies, such as cultivation. The research results of this paper show that green entrepreneurial motivation can significantly improve green entrepreneurial intentions. Besides, when psychological factors are considered, the results indicate that ecological values have an effect on green entrepreneurial intentions *via* green entrepreneurial motivation. However, there is no significant relationship between these two variables, indicating that, while those with motivation are more likely to have intentions, there are still some differences between them. Therefore, when considering the issues of cultivation and publicity, we should consider not only the factors that can directly improve green entrepreneurial intentions themselves, but also the factors that have an effect on green entrepreneurial motivation, such as the ecological values studied in this paper.

At the same time, the impact of major emergencies, such as COVID-19 on entrepreneurship, especially green entrepreneurship, should be viewed dialectically. It is often deemed that major emergencies may lead to less entrepreneurial intentions. However, because the survey data for this article were collected post-epidemic, the results of epidemic prevention and control have resulted in the intention of green entrepreneurship *via* the psychological factors discussed in this article, implying that the impact of major emergencies on entrepreneurship should be viewed differently. On the one hand, the economic downturn that has occurred in the aftermath of emergencies has rendered some small and micro businesses unsustainable. It may have an effect on potential entrepreneurs’ entrepreneurial intentions, but on the other hand, the prudent management and orderly recovery of major emergencies will also encourage potential entrepreneurs to have a more optimistic view of the overall entrepreneurial environment. On the subject of green entrepreneurship, significant events, such as COVID-19, which are already concerned with the ecological environment, can bolster the green entrepreneurial intentions of potential green entrepreneurs from a variety of perspectives, including ecological values. Our study applies the discussion of green entrepreneurial intention to the contemporary environment, focusing on the impact of emergencies, such as COVID-19 on green entrepreneurial intention. We used innovative structural equations to test hypotheses and discussed the relationship between COVID-19, psychological factors, and green entrepreneurial intention, as well as providing methodological guidance.

This study has some limitations but also identifies areas for future research. Firstly, this study examined the impact of the psychological factors, such as green entrepreneurial self-efficacy, optimism, and personality traits of green entrepreneurs, on green entrepreneurial intentions in the context of COVID-19 and explored the mediating role of green entrepreneurial motivation. Since this study uses undergraduate, postgraduate, and doctoral students from several Chinese universities as research subjects, the sample size is small and the scope of the sample is limited. Therefore, we strongly recommend that future researchers use these indicators to conduct longitudinal design studies on a more comprehensive samples, thus contributing to the literature on green entrepreneurship. We also suggest that future researchers may consider other factors that may affect green entrepreneurial intentions, such as background, income, risk-taking tendency, and social support, on the basis of the psychological factors selected in this paper. And furthermore, future researchers may employ these structures in a variety of samples. In the long run, realistic motivations for green entrepreneurship may be demonstrated to be a more dynamic process over time, characterized by constant adjustment and mutual optimization in response to changes in internal and external factors. These issues will pose significant obstacles to the upcoming study.

## Data Availability Statement

The original contributions presented in the study are included in the article/supplementary material, further inquiries can be directed to the corresponding author.

## Ethics Statement

Ethical review and approval was not required for the study on human participants in accordance with the local legislation and institutional requirements. The informed consent of the participants was implied through survey completion.

## Author Contributions

QC conceptualized and designed the empirical research and wrote the manuscript. WW took charge in designing the empirical research, collected the data, and wrote the manuscript. YZ designed the empirical research and performed the data analysis. CZ, YM, and ZP performed the data analysis and revised the manuscript. All authors worked collectively and significantly contributed to this manuscript.

## Funding

The work described in this manuscript was supported by the Sichuan Normal University’s School-level Special Funding Project for “Research and Interpretation of the Spirit of the Fourth Plenary Session of the 19th Central Committee of the Communist Party of China” (Research on the Coupling and Coordination of Industrial and Ecological and Green Governance Mechanism in Sichuan Tibetan Areas) (SNU19J4Z2019-14), the Sichuan Soft Science Research Project (2019JDR0345), the 2020 Sichuan International Science and Technology Innovation Cooperation Project (2020YFH0160), and the Social Science Planning Project of Sichuan Province (SC19TJ026 and SC21B097).

## Conflict of Interest

The authors declare that the research was conducted in the absence of any commercial or financial relationships that could be construed as a potential conflict of interest.

## Publisher’s Note

All claims expressed in this article are solely those of the authors and do not necessarily represent those of their affiliated organizations, or those of the publisher, the editors and the reviewers. Any product that may be evaluated in this article, or claim that may be made by its manufacturer, is not guaranteed or endorsed by the publisher.

## Appendix

**TABLE A1:** Questionnaire.

Characteristics of Study Participants	
1	Your gender: Male/Female
2	Your age: no more than 20/21–25/more than 26
3	Your current educational background: (during or completed) college for professional training/(during or completed) Bachelor Degree Study/(during or completed) Master Degree Study (Non-MBA)/(during or completed) MBA Study/(during or completed) Doctoral Degree Study
**Green Entrepreneurial Self-efficacy**	
1	I believe that if I do it with my heart, I can contribute to the environment.
2	I can find a way to help solve environmental problems.
3	Solving environmental problems is a contribution that each of us can make.
**Optimism**	
1	When the situation is uncertain, I always expect good results.
2	I think people in the majority of our society are good.
3	Most of the time, I am high-spirited.
**Ecological Values**	
1	I am willing to give out part of the income if I am sure that the money can be used to prevent and control environmental pollution.
2	Environmental protection takes precedence over economic growth, even if it may slow down economic growth or increase unemployment rate.
3	I think the current ecological and environmental problems are serious (greenhouse effect, destruction of biodiversity, water pollution, etc.).
**Sense of Social Responsibility**	
1	College students should not only follow the social ethics, but also lead them.
2	Being shirtless, wearing slippers, and smoking in public places will bring negative influence to the city and public environment.
3	When important group activities conflict with my personal ones, I tend to participate in group activities actively.
4	After a major disaster, I tend to donate money or useful goods.
**Green Entrepreneurial Motivation**	
1	I would like to start a green enterprise in order to solve employment problems.
2	I would like to start a green enterprise in order to be recognized by the society.
3	I would like to start a green enterprise in order to make a profit.
4	I would like to start a green enterprise in order to help stimulate the national economy.
5	I would like to start a green enterprise in order to make a contribution to social development.
6	I would like to start a green enterprise in order to contribute to the development of the ecological environment.
**Green Entrepreneurial Intentions**	
1	I am very interested in green entrepreneurship.
2	I have seriously considered things about green entrepreneurship.
3	I will try my best to start my own green enterprise.
4	I am preparing for green entrepreneurship in the future.
5	I firmly believe that I will establish a green enterprise in the future.
**COVID-19 Perception**	
1	COVID-19 has affected my confidence in my (green) entrepreneurship.
2	COVID-19 has affected my career (including studies, work, etc.).
3	During COVID-19, I feel nervous, anxious or restless.
4	During COVID-19, I tend to worry too much.
5	During COVID-19, I tend to be more irritable in some ways.
