# Corrigendum: COVID-19 to Green Entrepreneurial Intention: Role of Green Entrepreneurial Self-Efficacy, Optimism, Ecological Values, Social Responsibility, and Green Entrepreneurial Motivation

**DOI:** 10.3389/fpsyg.2022.808812

**Published:** 2022-03-07

**Authors:** Wenke Wang, Qilin Cao, Chaoyang Zhuo, Yunhan Mou, Zihao Pu, Yunhuan Zhou

**Affiliations:** ^1^Business School, Sichuan Normal University, Chengdu, China; ^2^Business School, Sichuan University, Chengdu, China; ^3^The People's Bank of China School of Finance, Tsinghua University, Beijing, China; ^4^School of Public Health, Yale University, New Haven, CT, United States; ^5^Department of Statistics and Actuarial Science, The University of Hong Kong, Pokfulam, Hong Kong, SAR China

**Keywords:** green entrepreneurial intention, COVID-19, self-efficacy, optimism, ecological values, social responsibility, entrepreneurial motivation

In the original article, there was an error in the Funding statement as published. Instead of “School-level special project of Sichuan Normal University,” the correct name for the Funder is “Sichuan Normal University's School-level Special Funding Project for “Research and Interpretation of the Spirit of the Fourth Plenary Session of the 19th Central Committee of the Communist Party of China” (Research on the Coupling and Coordination of Industrial and Ecological and Green Governance Mechanism in Sichuan Tibetan Areas)”. The funder “National Social Science Foundation of China (21BGL189)” has also been removed from the statement. The corrected Funding statement is shown below.

## Funding

The work described in this manuscript was supported by the Sichuan Normal University's School-level Special Funding Project for “Research and Interpretation of the Spirit of the Fourth Plenary Session of the 19th Central Committee of the Communist Party of China” (Research on the Coupling and Coordination of Industrial and Ecological and Green Governance Mechanism in Sichuan Tibetan Areas) (SNU19J4Z2019-14), the Sichuan Soft Science Research Project (2019JDR0345), the 2020 Sichuan International Science and Technology Innovation Cooperation Project (2020YFH0160), and the Social Science Planning Project of Sichuan Province (SC19TJ026 and SC21B097).

The authors apologize for these errors and state that they do not change the scientific conclusions of the article in any way. The original article has been updated.

## Publisher's Note

All claims expressed in this article are solely those of the authors and do not necessarily represent those of their affiliated organizations, or those of the publisher, the editors and the reviewers. Any product that may be evaluated in this article, or claim that may be made by its manufacturer, is not guaranteed or endorsed by the publisher.

